# Preclinical Research on a Mixture of Red Ginseng and Licorice Extracts in the Treatment and Prevention of Obesity

**DOI:** 10.3390/nu12092744

**Published:** 2020-09-09

**Authors:** Yulong Zheng, Eun-Hye Lee, Ji-Hyun Lee, Gyo In, JongHan Kim, Mi-Hyang Lee, Ok-Hwan Lee, Il-Jun Kang

**Affiliations:** 1 Department of Food Science and Nutrition, Hallym University, Chuncheon 24252, Korea; zyl1994@naver.com (Y.Z.); eunhye@hallym.ac.kr (E.-H.L.); leejihyun@hallym.ac.kr (J.-H.L.); 2 The Korean Institute of Nutrition, Hallym University, Chuncheon 24252, Korea; 3 Korea Ginseng Corporation Research Institute, Korea Ginseng Corporation, Daejeon 34337, Korea; 20109042@kgc.co.kr (G.I.); bellone@kgc.co.kr (J.K.); mhlee@kgc.co.kr (M.-H.L.); 4 Department of Food Science and Biotechnology, Kangwon National University, Chuncheon 24341, Korea; loh99@kangwon.ac.kr

**Keywords:** anti-obesity, extract mixture, dose-dependent, preclinical

## Abstract

The anti-obesity effects of RL (a 3:1 mixture of *Panax ginseng* saponin fractions and *Glycyrrhiza glabra* L. extracts) on 3T3-L1 adipocytes and C57BL/6J obese mice were evaluated at different concentrations. We investigated the anti-obesity effects of RL through lipid accumulation inhibition rate, serum lipid composition analysis, adipose tissue size, adipogenic transcription factors and AMPK pathway. RL inhibited the lipid accumulation of 3T3-L1 adipocytes in a dose-dependent manner at concentrations of 50–200 μg/mL without cytotoxicity (50–400 μg/mL). Oral administration of RL at the highest concentration (400 mg/kg/day) did not cause significant liver toxicity in high-fat diet-induced obese mice. RL stimulated adiponectin secretion in a dose-dependent manner and primarily mediates the AMPK pathway to inhibit triglyceride synthesis and attenuate adipocyte hypertrophy. RL significantly reduced weight in obese mice, but none of the body weight, adipose tissue weight, serum triglyceride level, and AMPK pathway activation degree showed any difference between dosing concentrations of 200 and 400 mg/kg/day. Therefore, 200 mg/kg/day of RL is the optimal preclinical concentration, which can be a reference concentration for conversion into a human clinical trial dose.

## 1. Introduction

Obesity is a serious challenge facing contemporary society. Being overweight or obese increases the incidence of Type 2 diabetes, hypertension, hyperlipidemia, non-alcoholic fatty liver and cancer [1]. Obesity causes not only various metabolic syndromes, but also social and economic problems [2]. Thereby, researchers around the world are actively seeking effective methods or substances to treat or improve obesity [3,4,5]. As one highly investigated strategy, there are now countless studies on natural products to improve obesity [6,7,8].

Hence the use of natural foods or natural medicines to treat obesity is likely to be an important focus going forward [9] and significantly, is supported by the World Health Organization [10]. The advantages of natural products in treating or preventing obesity are safety and low side effects [11]. Among various natural products, red ginseng (*Panax ginseng*) and licorice (*Glycyrrhiza glabra* L.) have numerous biologic activities as traditional medicines and new functional foods [12,13]. There are many formulas in Traditional Korean Medicine and Traditional Chinese Medicine that contain a combination of ginseng and licorice. For example, Sijunzi decoction with ginseng as the main ingredient and licorice as the auxiliary part has the effects of relieving cramps, pain-relief and curing gastric ulcers [14]. In addition, medicinal plant decoctions Chu-Yeh-Shih-Kao-Tang [15], Bai-Hu-Tang [16] and Byakko-ka-Ninjin-to [17] can improve insulin sensitivity and lower blood glucose level; Chai-Ling-Tang has immunomodulatory effects [18]; Lizhong Wan helps digestive system diseases [19], all the above preparations more or less contain ginseng and licorice combined. According to statistics, ginseng has the highest frequency among the herbs that are often used in combination with licorice [20].

Both red ginseng saponins and licorice extract (LE) have been reported to improve obesity symptoms [21,22]. When the proportion of ginseng in the combination of ginseng and licorice is higher than 50%, it will increase cell permeability [23]. Therefore, we use red ginseng extract (RE) as the primary material and mixed RE and LE at a 3:1 mass ratio and named RL. This study evaluated the dose-dependent anti-obesity efficacy of RL at different concentrations and briefly discussed its mechanism of action. These results will supply a theoretical basis for the concentration of RL to be used in subsequent clinical trials to develop into functional foods that can be practically applied.

## 2. Materials and Methods

### 2.1. Materials

All reagents used in the experiment were guaranteed reagent grade and HPLC-grade. Acetonitrile, ethanol and methanol were from Merck (Darmstadt, Germany). Isopropanol (100%) was from J.T. Baker Chemical (Phillipsburg, NJ, USA). Phosphate-buffered saline (PBS) was from Lonza (Walkersville, MD, USA). Dulbecco’s modified Eagle’s medium (DMEM) was from BioWest (Riverside, MO, USA). Paraformaldehyde (4%) was from Biosesang (Seongnam-si, Korea). Bovine calf serum (BCS), fetal bovine serum (FBS), penicillin/streptomycin (P/S) and insulin were from Gibco (Grand Island, NY, USA). Ginsenoside Rg_1_, Rb_1_ and Rg_3_(S) were from ChromaDex Co. (Irvine, CA, USA). Dexamethasone (Dex), 3-isobutyl-1-methylxanthine (IBMX), thiazolyl blue tetrazolium bromide (MTT), dimethyl sulfoxide (DMSO), Oil Red O, glycyrrhizin, 2-methyl-2-butanol and 2,2,2-tribromoethanol (Avertin) were from Sigma-Aldrich (St. Louis, MO, USA). RIPA buffer and bicinchoninic acid (BCA) protein assay kit were from Thermo Fisher Scientific (Waltham, MA, USA). Primary antibodies, anti-rabbit β-actin, PPARγ, C/EBPα, adiponectin, AMPK, p-AMPK, ACC, p-ACC were from Cell Signaling Technology (Danvers, MA, USA), SREBP-1c and CPT-1 from Santa Cruz Biotechnology (Dallas, TX, USA).

### 2.2. Preparation of Sample

RE and LE were provided by Korean Ginseng Corporation (Daejeon, Korea). Red ginseng extract (*Panax ginseng* saponin fractions) was fractionated with water, 30% ethanol and 95% ethanol as the mobile phase through Diaion HP20 column (Sigma, St. Louis, MO, USA) chromatography and the 95% ethanol fraction was retained. Dry licorice (*Glycyrrhiza glabra* L.) was repeatedly subjected to reflux extraction using 30% ethanol at 80 °C. These two crude extractions were decompressed, concentrated and spray-dried. The powdered sample (RL) was obtained by mixing RE and LE in a mass ratio of 3:1.

### 2.3. HPLC Analysis

HPLC analysis of RE and LE were performed using a Waters Alliance system equipped with a binary solvent delivery pump, an autosampler and a PDA detector (Waters Co., Milford, MA, USA). The output signal was monitored at a wavelength of 203 nm and 254 nm and processed using Empower 2 software. The chromatographic separation of RE was performed using a Hypersil GOLD^TM^ (5 μm, 4.6 × 250 mm, Thermo, Waltham, MA, USA), while the LE separation was performed using a Supelco Discovery C18 column (5 μm, 4.6 × 250 mm, Thermo, Waltham, MA, USA). The column temperature and autosampler tray temperature were maintained at 35 °C and 25 °C, respectively. The mobile phase used for RE and LE analysis consisted of acetonitrile (solvent A) and water (solvent B) at a flow rate of 1.6 mL/min and 1.0 mL/min, respectively. Gradient elution conditions are as follows: RE: 0–10 min (20% A), 40 min (35% A), 55 min (50% A), 70 min (65% A), 72–82 min (90% A) and 84–90 min (20% A); LE: 0–19 min (19% A), 35 min (50% A), 36 min (100% A) and 40–42 min (19% A).

### 2.4. Cell Culture and Differentiation

3T3-L1 preadipocytes (American Type Culture Collection, CL-173, Manassas, VA, USA) were plated into 24-well plates at a density of 5 × 10^4^ cells/well and grown in DMEM containing 10% BCS and 1% P/S (37 °C, 5% CO_2_). When cells reached confluence they were induced to differentiate by MDI (0.5-mM IBMX, 1-μM Dex and 10-μg/mL insulin) differentiation DMEM containing 10% FBS and 1% P/S. RL was added at different concentrations (50, 100, 200 and 400 μg/mL) whenever the medium was changed.

### 2.5. Cell Viability

MTT assay was used to test the viability of 3T3-L1 preadipocytes. Preadipocytes were treated with different concentrations of RL (50, 100, 200 and 400 μg/mL) for 24 h. Then 200 μL of MTT (2 mg/mL) was added to the medium and incubated for 2 h. After removing the medium, formazan salt was dissolved in DMSO and absorbance measured at a wavelength of 570 nm using a UV-visible spectrometer (Multiskan FC, Thermo Fisher Scientific, Inc., Waltham, MA, USA).

### 2.6. Lipid Accumulation Assay

Lipid accumulation of 3T3-L1 adipocytes was measured by Oil red O staining. Preadipocytes induced differentiation into mature adipocytes (Day 8) and then the medium was removed. Cells were rinsed with PBS and fixed in 4% paraformaldehyde for 1 h at room temperature. The fixed cells were stained with Oil Red O for 20 min at room temperature and then washed with distilled water. The Oil red O was then eluted with 100% isopropanol, and the absorbance was measured at a wavelength of 520 nm using a UV-visible spectrometer.

### 2.7. Mice and Diets

Five-week-old male C57BL/6J mice (Central Laboratory Animal, Inc., Seoul, Korea) were subjected to a one-week environmental adaptation (temperature 24 ± 2 °C, relative humidity 55 ± 5% and 12 h alternating dark:light cycles). Mice were randomly divided into the following seven groups (*n* = 10/group) after induction of obesity with a high-fat diet (HFD; 60% kcal fat, Envigo, Madison, WI, USA) for two weeks: (i) normal-fat diet (NFD; 10% kcal fat, Envigo, Madison, WI, USA) group, (ii) HFD group, (iii) GC200 group (HFD + 200 mg/kg of *Garcinia cambogia* water extract (GC)) as a positive control [24], (iv) RL50 group (HFD + 50 mg/kg of RL), (v) RL100 group (HFD + 100 mg/kg of RL), (vi) RL200 group (HFD + 200 mg/kg of RL), (vii) RL400 group (HFD + 400 mg/kg of RL). The samples were dissolved in tap water and orally administered to mice for 8 weeks once per day. All experimental animals were allowed free access to food and water. Bodyweight, food intake and water intake were measured weekly. At the end of the experiment, the mice were fasted for 12 h and anesthetized with avertin. Blood was taken from the orbital vein and centrifuged (3000× *g* for 15 min at 4 °C; centrifuge 5424R, Eppendorf, Hamburg, Germany.) to obtain serum, which was stored at −70 °C. Measurement of serum indicators was by automated clinical chemistry analyzer (FUJI DRI-CHEM NX500i, Tokyo, Japan), as reported previously [25]. The experimental protocol was approved by the Institutional Animal Care and Use Committee (IACUC) of Hallym University (Approval number: Hallym 2018-61).

### 2.8. Histological Analysis

After mice were sacrificed by cervical dislocation, the organs were weighed and rinsed with physiological saline. A portion of the epididymal adipose tissue was fixed in 4% paraformaldehyde for 48 h at 4 °C. The remaining organs were stored at −70 °C. Fixed epididymal adipose tissue was dehydrated by an automated tissue processor (TP1020; Leica Biosystems, Nussloch, Germany) using a series of graded ethanol solutions, embedded in paraffin and cut into 8-μm tissue sections. Sections were stained with hematoxylin and eosin (H&E), and adipose tissue cell morphology was recorded by optical microscopy. The size of adipose tissue cells was estimated by Adiposoft software (National Institutes of Health, Bethesda, MD, USA).

### 2.9. Immunoblot Assay

Immunoblot analysis performed as previously described [25]. Briefly, proteins from samples were extracted with RIPA buffer. After the total protein content was determined by BCA quantification, 10 μg of protein was loaded and separated by SDS-PAGE. Proteins were transferred to PVDF membranes and incubated with primary antibody overnight at 4 °C and then incubated with the secondary antibody for 1 h at room temperature. The primary antibody and secondary antibody were diluted in 5% BSA solution at the ratio of 1:1000 and 1:10,000, respectively.

### 2.10. Statistical Analysis

SPSS software (25.0, Statistical Package for Social Science, Inc.) was used for statistical analysis. The significance was calculated using one-way analysis of variance (ANOVA) and considered statistical significance at *p* < 0.05.

## 3. Results

### 3.1. Composition of RE and LE

**Figure 1:**
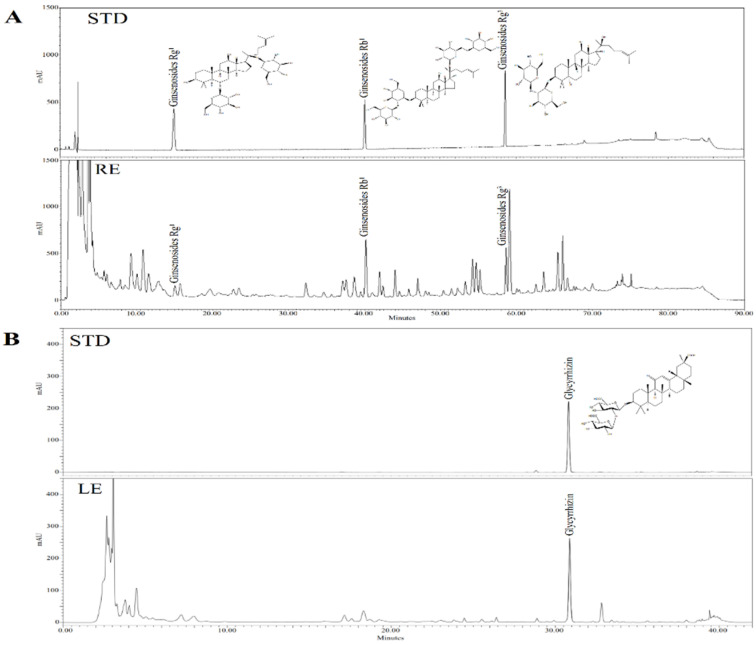
HPLC chromatograms of major compounds of (**A**) RE and (**B**) LE. RE—red ginseng extract; LE—licorice extract; STD—standard compounds. Identification of major compounds was determined by comparing to retention times of the STD.

HPLC analysis of RE and LE (Figure 1) showed that the primary ginsenosides in RE were Rg_1_, Rb_1_ and Rg_3_(S) (total content of 40.1 mg/g). The main compound in LE was glycyrrhizin with a content of 27.3 mg/g.

### 3.2. High Concentrations of RL Did Not Cause Cytotoxicity

**Figure 2:**
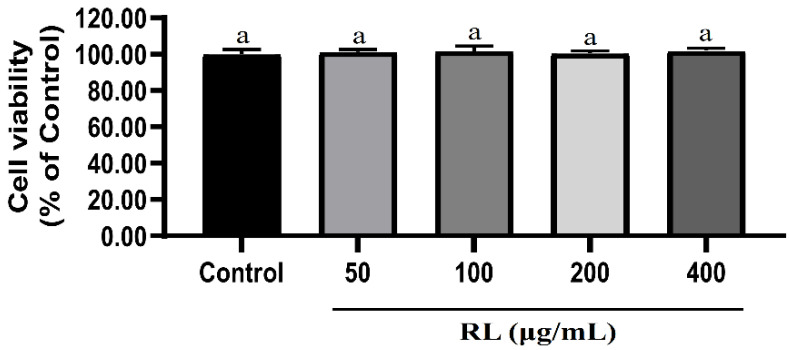
Effects of RL at various concentrations on cell viabilities of 3T3-L1 preadipocytes. Values presented as mean ± SD of experiments (*n* = 3). Control—undifferentiated induced 3T3-L1 preadipocytes; RL—red ginseng and licorice extracts mixed at a 3:1 mass ratio. In Duncan’s multiple range test, *p* < 0.05 expressed in different lowercase letters and *p* > 0.05 expressed in same lowercase letters.

None of the tested concentrations of RL (50–400 μg/mL) cause significant cell death or shedding (Figure 2). Even at the highest concentration (400 μg/mL), RL is nontoxic to cells.

### 3.3. Different Concentrations of RL Have Different Inhibitory Rates on Lipid Accumulation

**Figure 3:**
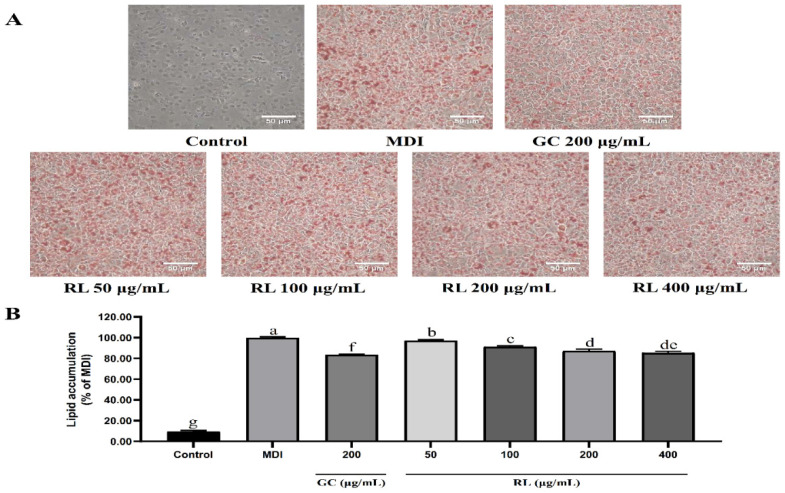
Inhibitory effects of RL at various concentrations on lipid accumulation in adipogenesis of 3T3-L1 cells. Control—undifferentiated induced 3T3-L1 preadipocytes; MDI—differentiated 3T3-L1 adipocytes induced by 0.5-mM IBMX, 1-μM Dex and 10-μg/mL insulin; GC—*Garcinia cambogia* water extract; RL—red ginseng and licorice extracts mixed at a 3:1 mass ratio. Values presented as mean ± SD of experiments (*n* = 3). (**A**) Post-confluent 3T3-L1 preadipocytes were treated with each extract to accumulate lipids after 8 days of differentiation; (**B**) oil red O-staining on Day 8. In Duncan’s multiple range test, *p* < 0.05 expressed in different lowercase letters and *p* > 0.05 expressed in same lowercase letters.

Based on Oil red O staining of the MDI differentiated and control groups, it was confirmed that preadipocytes had differentiated entirely into mature adipocytes (Figure 3). RL showed significant inhibition of lipid accumulation in a dose-dependent manner (50–200 μg/mL) compared to the MDI alone group. Lipid inhibition rates at RL concentrations of 200 and 400-μg/mL were 12.6% and 14.4%, respectively, which was not significantly different.

### 3.4. Suppression of Adipogenic and Lipogenic Transcription Factors by Different Concentrations of RL

**Figure 4:**
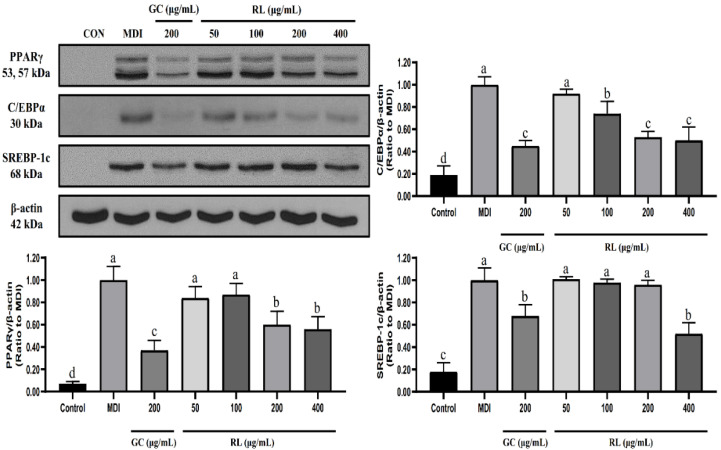
Effects of RL at various concentrations on the expression levels of 3T3-L1 adipocyte transcription factors. Control—undifferentiated induced 3T3-L1 preadipocytes; MDI—differentiated 3T3-L1 adipocytes induced by 0.5-mM IBMX, 1-μM Dex and 10-μg/mL insulin; GC—*Garcinia cambogia* water extract; RL—red ginseng and licorice extracts mixed at a 3:1 mass ratio. Western blot analysis of PPARγ, C/EBPα and SREBP-1c was performed on Day 8 of cell differentiation. Values presented as mean ± SD of experiments (*n* = 3). In Duncan’s multiple range test, *p* < 0.05 expressed in different lowercase letters and *p* > 0.05 expressed in same lowercase letters.

As shown in Figure 4, RL significantly reduced PPARγ (peroxisome proliferator-activated receptor-gamma) expression at a concentration of 200-μg/mL compared to MDI but did not change further between 200 and 400 μg/mL. RL also showed a dose-dependent decrease in C/EBPα (CCAAT/enhancer-binding protein alpha) expression at concentrations of 50 to 200 μg/mL. Moreover, RL significantly reduced the expression level of SREBP-1c (sterol regulatory element-binding protein 1c) however, only at a concentration of 400 μg/mL.

### 3.5. RL Can Improve Various Physiological Indicators of Obese Mice

Changes in bodyweights of mice are shown in Table 1. After initiating a high-fat diet, bodyweight in the HFD group increased by 1.47-fold compared to the NFD group. However, RL significantly reduced weight gain induced by a high-fat diet when taken orally at concentrations above 200 mg/kg/day. However, there was no significant difference in final bodyweight and weight gain among GC200, RL200 and RL400 groups.

There was no effect of RL at any concentration on food and water intake of mice (Table 2). Meanwhile, although food efficiency ratio (FER) tends to decrease with increasing RL concentration, there was no significant difference among the groups.

The organ weights of mice are shown in Table 3. There was no significant difference in kidney and spleen weights among the experimental groups. Liver, epididymal fat and visceral fat in the HFD group was 1.49-, 3.69- and 5.18-fold heavier than in the NFD group, respectively. RL at concentrations of 100–400 mg/kg/day began to reduce liver weight by 22%, 27% and 24%, respectively, compared with the HFD group. However, there was no statistical difference in liver weights among the GC200, RL100, RL200 and RL400 groups. Similar to weight change trends compared to the HFD group, RL at concentrations of 200 and 400 mg/kg/day significantly reduced the epididymal fat weight by 9.67% and 10.69%, respectively and the visceral fat weight decreased by 27% and 32%, respectively. There were no significant differences however, between GC200, RL200 and RL400 groups.

### 3.6. RL Attenuates Adipocyte Hypertrophy

**Figure 5:**
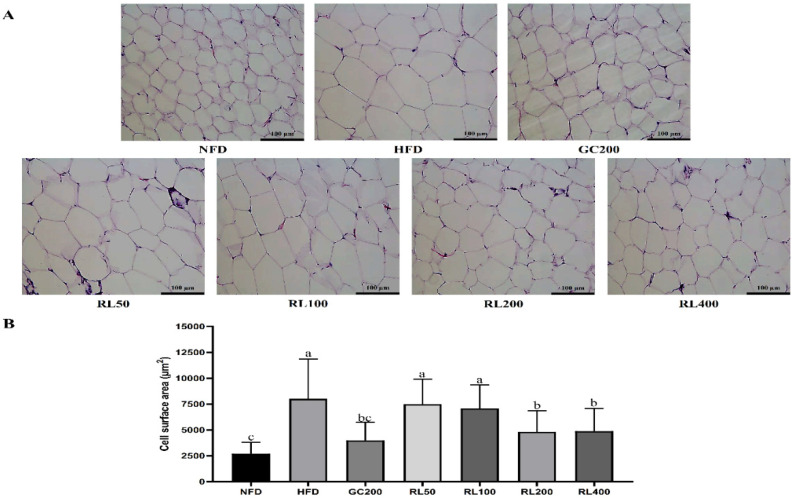
Effects of RL at various concentrations on the size of adipocytes in epididymal adipose. (**A**) hematoxylin–eosin staining of epididymal adipose tissue; (**B**) stained adipose tissue cell area estimated by Adiposoft software. RL—red ginseng and licorice extracts mixed at a 3:1 mass ratio; NFD—mice fed normal-fat diet (10% kcal fat); HFD—mice fed high-fat diet (60% kcal fat); GC200—HFD + 200 mg/kg/day of *Garcinia cambogia* water extract; RL50—HFD + RL at a concentration of 50 mg/kg/day; RL100—HFD + RL at a concentration of 100 mg/kg/day; RL200—HFD + RL at a concentration of 200 mg/kg/day; RL400—HFD + RL at a concentration of 400 mg/kg/day. In Duncan’s multiple range test, *p* < 0.05 expressed in different lowercase letters and *p* > 0.05 expressed in same lowercase letters.

Changes in adipocyte sizes are shown in Figure 5. HFD epididymal adipocytes were hypertrophic compared to the NFD group. As the concentration of RL increases, more cells can be observed under the same level of microscopic magnification. The size of adipocytes was reduced for all administered groups, but there appears to be no difference among the GC200, RL200 and RL400 groups.

### 3.7. RL Can Reduce Blood Lipids in Obese Mice

Serum parameters are shown in Table 4. An increase in RL concentration did not cause significant changes in serum alanine aminotransferase and aspartate aminotransferase. Compared with the HFD group, RL at concentrations of 200 and 400 mg/kg/day significantly decreased serum triglyceride levels by 36% and 44%, respectively. However, there was no significant difference between the RL400 and RL200 groups. The remaining serum indicators did not differ among groups.

### 3.8. RL Activates the AMPK Pathway in a Dose-Dependent Manner

**Figure 6:**
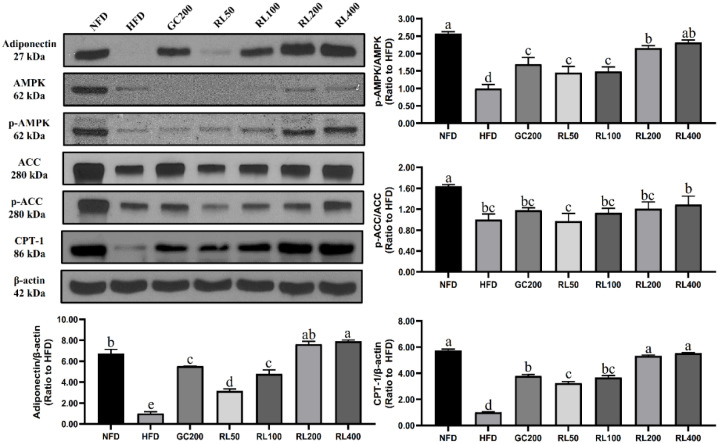
Effects of RL at various concentrations on energy metabolism-related proteins in epididymal adipose tissue of high-fat-diet-induced obese C57BL/6J mice. Values presented as mean ± SD of experiments (*n* = 3). RL—red ginseng and licorice extracts mixed at a 3:1 mass ratio; NFD—mice fed normal-fat diet (10% kcal fat); HFD—mice fed high-fat diet (60% kcal fat); GC200—HFD + 200 mg/kg/Day *Garcinia cambogia* water extract; RL50—HFD + RL at a concentration of 50 mg/kg/day; RL100—HFD + RL at a concentration of 100 mg/kg/day; RL200—HFD + RL at a concentration of 200 mg/kg/day; RL400—HFD + RL at a concentration of 400 mg/kg/day. In Duncan’s multiple range test, *p* < 0.05 expressed in different lowercase letters and *p* > 0.05 expressed in same lowercase letters.

As shown in Figure 6, RL significantly increased the expression of adiponectin which was reduced by HFD. It did so in a dose-dependent manner from 50–200 mg/kg/day. Consequently, RL significantly enhanced the extent of AMP-activated protein kinase (AMPK) phosphorylation compared to the HFD group, and dose dependence was only shown at 100–200 mg/kg/day. Although RL increased the phosphorylation of acetyl-CoA carboxylase (ACC), it did not differ significantly from the HFD group. In addition, RL significantly increased the expression of carnitine palmitoyltransferase I (CPT-1), but there was no difference between RL200 and RL400 groups.

## 4. Discussion

Researching new drugs or new functional foods will inevitably face the question of dose conversion from preclinical animal testing to human clinical trials [26]. Therefore, the maximum effective dose in the safe range is information that must be obtained [27]. One of the purposes of this study was to define the appropriate sample concentration in preclinical animal experiments for subsequent human trials.

RL did not exhibit any cytotoxicity even at 400 μg/mL, and it dose-dependently inhibited lipid accumulation in a range of 50–200 μg/mL. PPARγ, C/EBPα and SREBP-1c play crucial roles in adipocyte differentiation and lipid synthesis [28,29]. They are also key indicators for evaluating the anti-obesity activity of substances. RL significantly reduced PPARγ and C/EBPα expression at 200 μg/mL but was not further altered at 400 μg/mL. The only molecular change manifested by RL at 400 μg/mL was in reducing the expression of SREBP-1c. Therefore, we have reason to believe that the anti-lipid accumulation capacity of RL is not different between 200 μg/mL and 400 μg/mL.

The main ingredient of LE is glycyrrhizin (Figure 1B). Recently, other investigators demonstrated that herbal formulas with high levels of glycyrrhizin could mediate PPARγ and C/EBPα pathways to inhibit adipogenesis in 3T3-L1 adipocytes [30]. Based on the compositional analysis of RE, its main compounds are ginsenosides Rg_1_, Rb_1_ and Rg_3_ (Figure 1A). Ginsenosides Rg_1_ and Rg_3_ participate in PPARγ and AMPK pathways to inhibit lipid accumulation in 3T3-L1 adipocytes [31,32]. However, ginsenoside Rb_1_ promotes adipogenesis through PPARγ and C/EBPα pathways [33], which may cause the effects of ginsenosides Rg_1_ and Rg_3_ to be oppositional to Rb_1_. This seems to indicate that there is a certain antagonism in a specific concentration range, resulting in no significant change in transcription factors. Presumably, the balancing effects on these transcription factors at certain threshold concentrations may be the reason that there was no further difference in the inhibition of lipid accumulation in adipocytes between RL at 200 and 400 μg/mL (Figure 4).

In animal models, the active ingredients in extracts will usually be hydrolyzed in the intestine or metabolized by gut microbiota. Glycyrrhizinic acid has been reported to improve lipid deposition in obese rats [34]. However, the main pharmacological properties of LE are due to the 18β-glycyrrhetinic acid produced by the hydrolysis of glycyrrhizin in the intestine [35]. 18β-glycyrrhetinic acid can improve lipid metabolism disorders by inhibiting Type 1 cannabinoid receptor activity [36]. Most of the ginsenosides are metabolized by gut microbiota after they enter the body [37]. Ginsenosides produce bioactive metabolites after deglycosylation. The following processes are known to metabolize the three primary ginsenosides in RE: (i) The intestinal flora metabolizes Rg_1_ and after producing Rh_1_ and F_1_, it is eventually metabolized to 20-(S)-protopanaxatriol [38]; (ii) Rb_1_ is deglycosylated and converted to Rd, which is then metabolized to Rg_3_ and F_2_, respectively. F_2_ is further metabolized to Compound K, eventually producing 20-(S)-protopanaxadiol [39]; (iii) Rg_3_ is deglycosylated to Rh_2_ and then metabolized to 20-(S)-protopanaxadiol [40]. Among these parent compounds or metabolites, ginsenosides Rg_1_ [31], Rg_3_ [32], Rb_1_ [41], Rh_2_ [42], and Compound K [43] can activate the AMPK pathway.

AMPK is responsible for energy metabolism balance [44]. The activated AMPK pathway enhances energy production processes [45] and weakens energy consumption process [46]. Activated AMPK promoted phosphorylation of ACC to inhibit lipid synthesis. ACC phosphorylation reduced CPT-1 inhibitors, allowing fatty acids to β-oxidize. We have hypothesized that RL could improve lipid metabolism abnormalities in obese mice through the AMPK pathway, and this has now been partially confirmed. RL dose-dependently stimulated adiponectin expression in this study (Figure 6), but there was no difference between RL200 and RL400 groups. Upregulated expression of adiponectin activates AMPK phosphorylation in the RL200 group, but no further increase was seen in the RL400 group. Therefore, downstream proteins in the AMPK pathway (such as phosphorylated ACC and CPT-1) also showed similar trends. Through regulation of energy metabolism, the weight of liver and adipose tissue in obese mice was reduced. In addition, reduced serum triglycerides also attenuated the phenomenon of cell hypertrophy in adipose tissue (Figure 5 and Table 4). These results ultimately led to a significant weight loss of C57BL/6J obese mice in the RL200 and RL400 groups.

Through dose-dependency studies, oral administration of RL did not significantly change serum ALT and AST levels, but instead alleviated the hepatic lipotoxicity caused by a high-fat diet to a certain extent (Table 4). Kimura et al. [17] found that both ginseng and licorice can lower blood glucose, but a combination of ginseng and licorice will reduce their hypoglycemic effect. In this study, we also found that RL had no significant effect on the blood glucose levels of obese mice. Higher doses of RL, more than 400 μg/mL, may produce a more pronounced effect, however, which is limited in clinical applications due to the potential over uptake. We believe that we have found a rational anti-obesity mechanism for RL through transcription factors and AMPK pathways. However, the exact effect targets of RL were not determined due to the complex compositions of RL, which remains to be further studied.

## 5. Conclusions

In conclusion, RL has a dose-dependent anti-obesity effect in a particular concentration range. Based on the experimental data obtained, the concentration of RL used for the follow-up clinical trial can be converted from the optimal concentration of 200 mg/day, obtained from the preclinical experiments.

## Figures and Tables

**Table 1:** Effects of RL at various concentrations on initial body weight, final body weight and body weight gain in high-fat diet-induced obese C57BL/6J mice.

Parameter	NFD	HFD	GC200	RL50	RL100	RL200	RL400
Initial body weight (g)	21.63 ± 0.63 ^a^	21.61 ± 0.72 ^a^	21.51 ± 0.73 ^a^	21.56 ± 0.30 ^a^	21.58 ± 0.81 ^a^	21.69 ± 0.54 ^a^	21.50 ± 0.56 ^a^
Final body weight (g)	30.83 ± 1.54 ^c^	45.36 ± 2.53 ^a^	41.83 ± 2.47 ^b^	43.13 ± 2.32 ^a,b^	43.06 ± 4.52 ^a,b^	41.94 ± 2.53 ^b^	41.67 ± 3.77 ^b^
Body weight gain (g)	9.19 ± 1.65 ^c^	23.75 ± 2.41 ^a^	20.32 ± 2.39 ^b^	21.57 ± 2.34 ^a,b^	21.48 ± 4.46 ^a,b^	20.25 ± 2.32 ^b^	20.17 ± 3.81 ^b^

Values presented as mean ± SD of experiments (*n* = 10). RL—red ginseng and licorice extracts mixed at a 3:1 mass ratio; NFD—mice fed normal-fat diet (10% kcal fat); HFD—mice fed high-fat diet (60% kcal fat); GC200—HFD + 200 mg/kg/day of *Garcinia cambogia* water extract; RL50—HFD + RL at a concentration of 50 mg/kg/day; RL100—HFD + RL at a concentration of 100 mg/kg/day; RL200—HFD + RL at a concentration of 200 mg/kg/day; RL400—HFD + RL at a concentration of 400 mg/kg/day. In Duncan’s multiple range test, *p* < 0.05 expressed in different lowercase letters and *p* > 0.05 expressed in same lowercase letters.

**Table 2:** Effects of RL at various concentrations on food intake, water intake, energy intake and FER in high-fat diet-induced C57BL/6J mice.

Parameter	NFD	HFD	GC200	RL50	RL100	RL200	RL400
Food intake (g/day)	2.74 ± 0.13 ^a^	2.87 ± 0.12 ^a^	2.87 ± 0.22 ^a^	2.74 ± 0.15 ^a^	2.73 ± 0.23 ^a^	2.75 ± 0.13 ^a^	2.88 ± 0.10 ^a^
Water intake (g/day)	3.01 ± 0.42 ^a^	2.99 ± 0.57 ^a^	2.57 ± 0.54 ^a^	2.91 ± 0.46 ^a^	2.58 ± 0.40 ^a^	2.90 ± 0.42 ^a^	3.02 ± 0.51 ^a^
Energy intake (Kcal/day)	10.14 ± 0.49 ^b^	14.62 ± 0.61 ^a^	14.62 ± 1.13 ^a^	13.96 ± 0.78 ^a^	13.91 ± 1.18 ^a^	14.05 ± 0.65 ^a^	14.69 ± 0.51 ^a^
FER (%)	4.78 ± 0.82 ^b^	11.67 ± 3.07 ^a^	10.01 ± 2.06 ^a^	10.96 ± 2.34 ^a^	11.34 ± 1.99 ^a^	10.17 ± 1.52 ^a^	9.80 ± 1.77 ^a^

Values presented as mean ± SD of experiments (*n* = 10). RL—red ginseng and licorice extracts mixed at a 3:1 mass ratio; NFD—mice fed normal-fat diet (10% kcal fat); HFD—mice fed high-fat diet (60% kcal fat); GC200—HFD + 200 mg/kg/day of *Garcinia cambogia* water extract; RL50—HFD + RL at a concentration of 50 mg/kg/day; RL100—HFD + RL at a concentration of 100 mg/kg/day; RL200—HFD + RL at a concentration of 200 mg/kg/day; RL400—HFD + RL at a concentration of 400 mg/kg/day. Food efficiency ratio (FER) = body weight gain (g/day)/food intake (g/day). In Duncan’s multiple range test, *p* < 0.05 expressed in different lowercase letters and *p* > 0.05 expressed in same lowercase letters.

**Table 3:** Effects of RL at various concentrations on organ wet weight in high-fat diet-induced obese C57BL/6J mice.

Parameter	NFD	HFD	GC200	RL50	RL100	RL200	RL400
Liver (g)	1.02 ± 0.12 ^c^	1.52 ± 0.30 ^a^	1.19 ± 0.07 ^b,c^	1.24 ± 0.25 ^b^	1.18 ± 0.10 ^b,c^	1.10 ± 0.14 ^b,c^	1.15 ± 0.18 ^b,c^
Kidney (g)	0.27 ± 0.03 ^a^	0.30 ± 0.03 ^a^	0.29 ± 0.01 ^a^	0.29 ± 0.03 ^a^	0.30 ± 0.04 ^a^	0.29 ± 0.02 ^a^	0.29 ± 0.02 ^a^
Spleen (g)	0.06 ± 0.01 ^a^	0.06 ± 0.01 ^a^	0.06 ± 0.01 ^a^	0.06 ± 0.01 ^a^	0.06 ± 0.01 ^a^	0.06 ± 0.01 ^a^	0.06 ± 0.01 ^a^
Epididymal fat (g)	0.72 ± 0.16 ^c^	2.66 ± 0.23 ^a^	2.35 ± 0.20 ^b^	2.58 ± 0.27 ^a,b^	2.52 ± 0.21 ^a,b^	2.40 ± 0.10 ^b^	2.38 ± 0.21 ^b^
Visceral fat (g)	0.22 ± 0.09 ^c^	1.14 ± 0.35 ^a^	0.75 ± 0.23 ^b^	0.99 ± 0.44 ^a,b^	0.89 ± 0.35 ^a,b^	0.83 ± 0.25 ^b^	0.77 ± 0.37 ^b^

Values presented as mean ± SD of experiments (*n* = 10). RL—red ginseng and licorice extracts mixed at a 3:1 mass ratio; NFD—mice fed normal-fat diet (10% kcal fat); HFD—mice fed high-fat diet (60% kcal fat); GC200—HFD + 200 mg/kg/Day *Garcinia cambogia* water extract; RL50—HFD + RL at a concentration of 50 mg/kg/day; RL100—HFD + RL at a concentration of 100 mg/kg/day; RL200—HFD + RL at a concentration of 200 mg/kg/day; RL400—HFD + RL at a concentration of 400 mg/kg/day. In Duncan’s multiple range test, *p* < 0.05 expressed in different lowercase letters and *p* > 0.05 expressed in same lowercase letters.

**Table 4:** Effects of RL at various concentrations on serum lipid biomarkers in high-fat diet-induced obese C57BL/6J mice.

Parameter	NFD	HFD	GC200	RL50	RL100	RL200	RL400
ALT (U/L)	24.88 ± 5.51	69.75 ± 35.94	53.00 ± 19.03	48.75 ± 31.69	48.88 ± 16.63	40.38 ± 13.64	38.63 ± 20.30
AST (U/L)	77.63 ± 18.88 ^a^	97.88 ± 64.02 ^a^	78.50 ± 21.86 ^a^	70.50 ± 25.14 ^a^	73.38 ± 15.99 ^a^	72.75 ± 26.93 ^a^	64.50 ± 10.99 ^a^
GLU (mg/dL)	195.88 ± 43.04 ^b^	298.00 ± 44.14 ^a^	268.63 ± 47.44 ^a^	267.13 ± 55.78 ^a^	281.75 ± 45.39 ^a^	261.25 ± 33.65 ^a^	266.38 ± 31.63 ^a^
TG (mg/dL)	83.25 ± 24.56	94.00 ± 15.37	88.67 ± 17.12	77.38 ± 12.28	71.13 ± 18.93	60.13 ± 12.35 ^B^	52.63 ± 10.99 ^B,D^
TC (mg/dL)	122.25 ± 6.78 ^b^	181.88 ± 10.91 ^a^	180.25 ± 17.12 ^a^	175.88 ± 16.49 ^a^	179.88 ± 14.10 ^a^	172.25 ± 9.38 ^a^	170.38 ± 13.71 ^a^
HDL-C (mg/dL)	81.63 ± 12.40 ^b^	126.25 ± 18.91 ^a^	132.25 ± 11.89 ^a^	131.75 ± 9.74 ^a^	137.63 ± 14.57 ^a^	130.38 ± 12.14 ^a^	136.63 ± 9.75 ^a^
LDL-C (mg/dL)	16.35 ± 3.08	30.58 ± 9.00 ^A^	25.58 ± 2.51 ^A^	28.65 ± 9.92	28.03 ± 6.46 ^A^	27.18 ± 9.82	26.68 ± 4.45 ^A^

Values presented as mean ± SD of experiments (*n* = 10). ALT—alanine aminotransferases; AST—aspartate aminotransferases; GLU—glucose; TG—triglyceride; TC—total cholesterol; HDL-C—high-density lipoprotein cholesterol; LDL-C—low-density lipoprotein cholesterol; RL—red ginseng and licorice extracts mixed at a 3:1 mass ratio; NFD—mice fed normal-fat diet (10% kcal fat); HFD—mice fed high-fat diet (60% kcal fat); GC200—HFD + 200 mg/kg/day of *Garcinia cambogia* water extract; RL50—HFD + RL at a concentration of 50 mg/kg/day; RL100—HFD + RL at a concentration of 100 mg/kg/day; RL200—HFD + RL at a concentration of 200 mg/kg/day; RL400—HFD + RL at a concentration of 400 mg/kg/day. In Duncan’s multiple range test, *p* < 0.05 expressed in different lowercase letters and *p* > 0.05 expressed in same lowercase letters. In Dunnett’s T3 test, *p* < 0.05 expressed in capital letters: A—compared with NFD; B—compared with HFD; C—compared with GC200; D—compared with RL50; E—compared with RL100; F—compared with RL200; G—compared with RL400.
